# Activated platelets induce MLKL-driven neutrophil necroptosis and release of neutrophil extracellular traps in venous thrombosis

**DOI:** 10.1038/s41420-018-0073-2

**Published:** 2018-06-28

**Authors:** Daigo Nakazawa, Jyaysi Desai, Stefanie Steiger, Susanne Müller, Satish Kumar Devarapu, Shrikant R. Mulay, Takamasa Iwakura, Hans-Joachim Anders

**Affiliations:** 1 Medizinische Klinik und Poliklinik IV, Klinikum der Universität München, Munich, Germany; 2 Department of Rheumatology, Endocrinology and Nephrology, Faculty of Medicine and Graduate School of Medicine, Hokkaido University, Sapporo, Japan; 3 Pathologisches Institut, Ludwig-Maximilians Universität, München, Germany

**Keywords:** Immunopathogenesis, Necroptosis

## Abstract

Venous thromboembolic (VTE) disease, often manifesting as deep vein thrombosis or pulmonary embolism, involves clot formation consisting of blood cells and platelets locked in plasma protein and chromatin networks. The latter derives from neutrophil extracellular traps released by dying neutrophils; however, the molecular mechanisms of neutrophil death in VTE remains unknown. We speculated that mixed lineage kinase-like (MLKL)-driven neutrophil necroptosis contributes to VTE. Indeed, human inferior venous cava thrombus material stained positive for phosphorylated MLKL, the activated version of MLKL that executes necroptotic cell death. In mice, MLKL immunostaining showed co-localization of MLKL with citrullinated histone H3, a marker of neutrophil extracellular trap (NET) formation. These data provide indirect support for a role of MLKL-mediated necroptosis. As a functional proof, both the stabilizer of receptor-interacting protein kinase-1 (RIPK1) and necroptosis inhibitor necrostatin-1s as well as genetic deficiency of MLKL partially prevented clot formation upon inferior vena cava ligation in mice. In both experiments terminal deoxynucleotidyl transferase dUTP nick-end labeling, RIPK3, and citrullinated histone H3+ areas were markedly reduced within the remnant thrombus. In vitro, thrombin-activated platelets induced cell death and NET formation in human neutrophils, which was inhibited by necrostatin-1s treatment. Necrostatin-1s and necrosulfonamide also inhibited neutrophil–platelet aggregate formation induced by tumor necrosis factor-α but had no effect on platelet activation itself. We conclude that in VTE, activated platelets, and possibly other triggers, induce neutrophil necroptosis, a process contributing to clot formation by releasing chromatin in the extracellular space.

## Introduction

Venous thromboembolism (VTE) is a complication of multiple different medical conditions and a major cause of morbidity and mortality worldwide^1^. Although it can occur in any location of the venous system, it primarily manifests clinically as deep vein thrombosis (DVT) or pulmonary embolism^2^. Local microvascular venous thrombosis is common at sites of trauma or infections but occurs also in life-threatening systemic disease states as disseminated intravascular coagulation^3^. Endothelial dysfunction and the activation of coagulation factors in the plasma are central elements in clot formation, but the clot itself largely consists of cellular elements such as red blood cells, platelets, and neutrophils all contributing to clot formation^2^. Red blood cell-derived microvesicles or adenosine diphosphate (ADP) initiate thrombin generation and platelet activation, respectively^4,5^. The role of platelets in VTE is less prominent than in arterial thrombosis. Nevertheless, thrombocytosis has been attributed as risk factor for VTE^6^. Pathogens and danger-associated molecular patterns (DAMPs) stimulate neutrophils to activate the clotting system, an interaction referred to as immunothrombosis^7^. Neutrophils, themselves, contribute to clot formation by releasing neutrophil extracellular traps (NETs), that is, networks consisting of extracellular chromatin, cytoplasmic, and granular proteins as well as histones that elicit immunostimulatory and cytotoxic effects on microvascular endothelial cells^8^. Indeed, netting neutrophil, monocytes, and platelets cooperate to initiate and propagate VTE^9^. Currently, little is known about the molecular mechanisms of VTE-related NET formation. It is shown that platelets release high mobility group protein B1 (HMGB1), which indeed triggers NET formation^10,11^; however, the execution pathway of neutrophil death and chromatin release in this context remains unknown.

Recently, receptor-interacting protein kinase-3 (RIPK3), a protein involved in inflammation as well as regulated necrosis^12^, has been reported to promote platelet activation in arterial thrombosis^13^. Interestingly, RIPK3 is also expressed in neutrophils and contributes to crystal-induced and microparticle-induced NET formation^14,15^, a process associated with neutrophil death and is therefore named neutrophil necroptosis^16^. Necroptosis is a regulated form of cell necrosis involving necrosome formation by RIPK3 and the pseudokinase mixed lineage kinase domain-like (MLKL)^17–19^. Indeed, MLKL oligomers form pores into nuclear and plasma cell membranes facilitating cell necrosis and chromatin release into the extracellular space^20^. Thus, MLKL-driven neutrophil necroptosis may contribute to gout and other microparticle-triggered diseases involving NETs^16,21^, but its role in VTE is speculative. Here, we hypothesized that MLKL-dependent neutrophil necroptosis may contribute to VTE, and thus employed specific antagonists and *Mlkl*-deficient mice to address this concept experimentally in vitro and in vivo.

## Results

### Inferior vena cava thrombi of human and mouse stains positive for markers of necroptosis

**Fig. 1:**
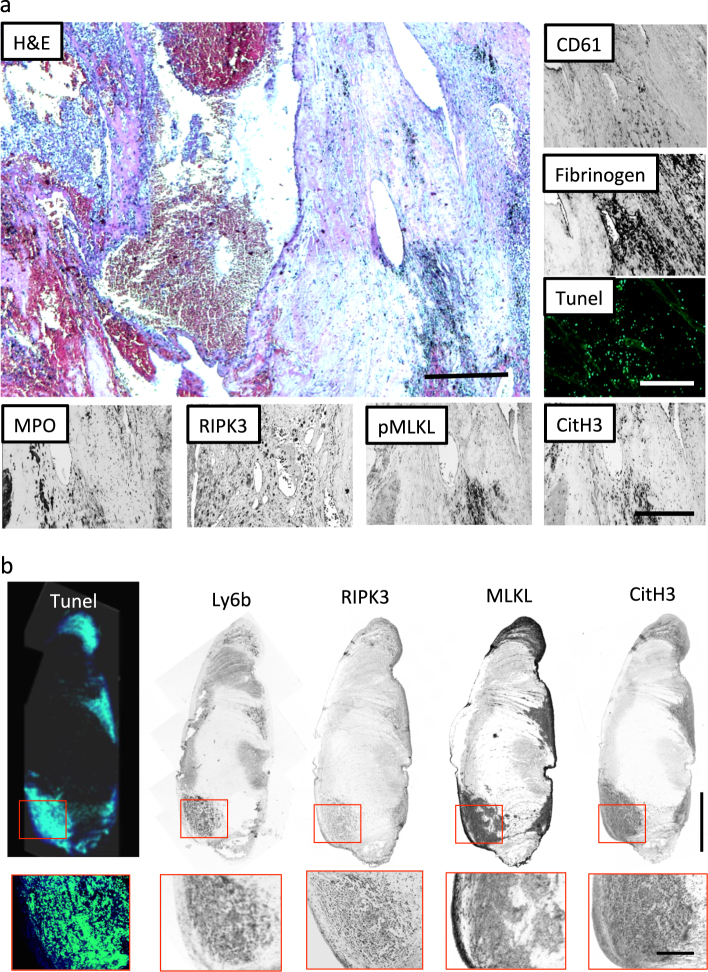
Programmed necrosis contributes to thrombus formation in human and mouse. **a** Paraffin-embedded sections of inferior vena cava (IVC) thrombus of patient with renal cell carcinoma (upper left panel). H&E staining shows leukocyte infiltration into the thrombus (upper right). Immunohistochemistry for CD61 and fibrinogen (lower panel). TUNEL, myeloperoxidase (MPO), receptor-interacting protein kinase-3 (RIPK3), and phosphorylated mixed lineage kinase-like (pMLKL) positive blood cells were detected in the thrombus. Scale bar = 500 μm. **b** Thrombus of mouse IVC ligation model. From the left panel, the staining shows TUNEL, Ly6b, RIPK3, MLKL, citrullinated histone-3 (CitH3). Upper figures show the whole thrombus (scale bar = 1 mm) and lower figures show magnified image (scale bar = 250 μm)

To examine whether RIPK/MLKL-dependent necroptosis is involved in thrombus formation, we performed immunostaining of an autopsy sample of a patient with inferior vena cava thrombus due to renal cell carcinoma. Hematoxylin and eosin (H&E) staining showed that infiltrating leukocytes were present in the thrombus along with CD61^+^ platelets and fibrinogen. The presence of dead cells inside the thrombus was identified by terminal deoxynucleotidyl transferase dUTP nick-end labeling (TUNEL) staining. In addition, immunostaining of myeloperoxidase (MPO) and citrulinated histone-3 (CitH3), as well as RIPK3 and phosphorylated MLKL, showed the presence of NETs with a suggestive involvement of MLKL activation (Fig. 1a). Next, we assessed the same parameters in a mouse model of inferior vena cava (IVC) thrombosis. IVC thrombus was induced in wild-type (C57BL/6N) male mice by ligation of IVC below the left renal vein without manipulating the side branches. At 72 h after surgery, thrombi developed in the IVC, in which infiltrated Ly6b^+^ leukocytes showed high expression of RIPK3-MLKL, CitH3, and TUNEL positivity (Fig. 1b). Therefore, we conclude that IVC thrombi of human and mouse stains positive for markers of necroptosis, NETs, and cell death.

### Pharmacological RIPK1 inhibition reduces clot size in murine IVC thrombosis

**Fig. 2:**
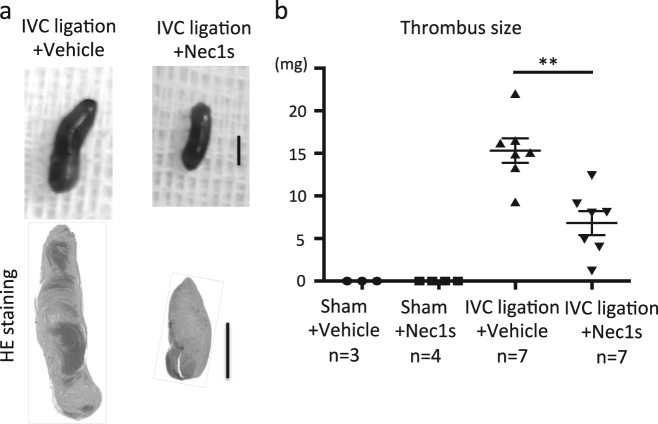
Necrostatin-1s (Nec1s) inhibits thrombus formation. The IVC of wild-type male mice (11–13 weeks old) was ligated under the anesthesia with pretreatment of vehicle (5% DMSO in PBS) or Nec1s, and all mice were sacrificed 3 days after the operation. **a** Left and right image show the thrombus in IVC ligation model with vehicle and Nec1s, respectively. Upper photos show macroscopic findings and lower figures show H&E staining. Scale bar = 1 mm. **b** The graph shows the thrombus size of sham-operated mice with vehicle (*n* = 3) or Nec1s (*n* = 4), and IVC-ligated mice with vehicle (*n* = 7) or Nec1s (*n* = 7). Data are mean ± SEM in each group. **p < 0.01 vs. respective control

**Fig. 3:**
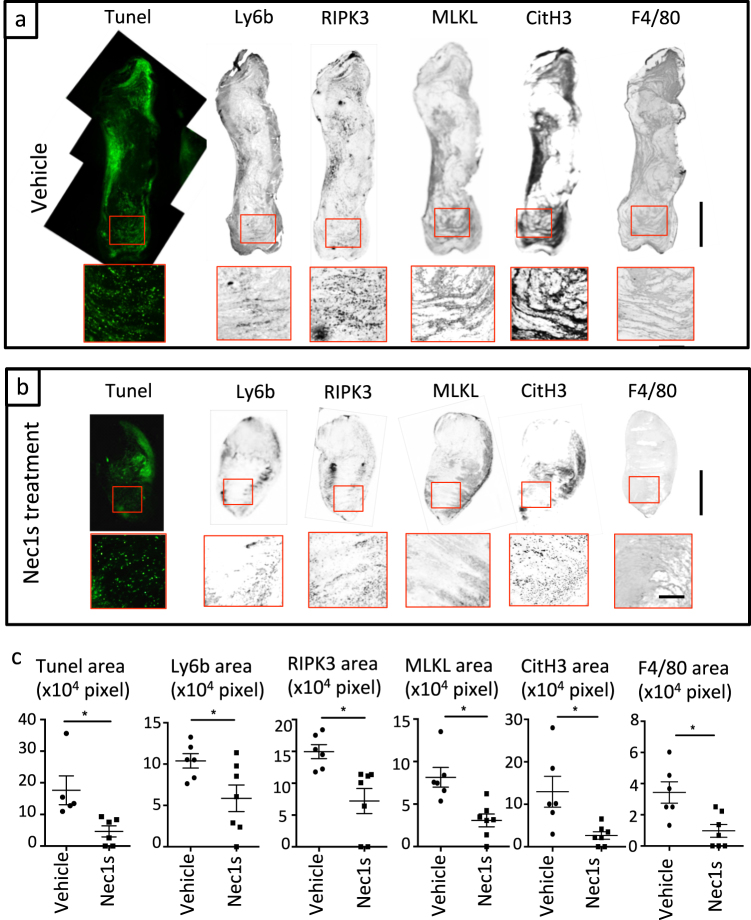
Nec1s suppresses necroptosis and NET-related signaling pathway during thrombus formation. **a** Representative figures of IVC-ligated mice with pretreatment of vehicle and **b** Nec1s. From left panel, the staining shows TUNEL, Ly6b, RIPK3, MLKL, CitH3, and F4/80. Upper figures show the whole thrombus (scale bar = 1 mm) and lower figures show magnified image (scale bar = 250 μm). **c** Quantification of positive area of each staining. Data are mean ± SEM in each group. *p < 0.05 vs. respective control

To assess whether necroptosis signaling contributes to clot formation, we evaluated the effect of pharmacological inhibition of necroptosis with the RIPK1 stabilizer necrostatin-1s (Nec1s) in the aforementioned IVC ligation venous thrombosis model. Wild-type mice were pre-treated prior to IVC ligation with Nec1s. Macroscopic findings revealed that Nec1s treatment significantly reduced clot formation (measured as thrombus weight) after IVC ligation (Fig. 2). TUNEL staining showed that Nec1s treatment reduced cell death inside thrombi compared to vehicle (Fig. 3a, b). Furthermore, the number of infiltrating Ly6b^+^ granulocytes in thrombi of Nec1s-treated mice were significantly lower than in controls. The expression of RIPK3-MLKL and CitH3 in thrombi was mainly co-localized with blood cells and the overexpression was suppressed by Nec1s treatment (Fig. 3a–c). Flow cytometric analysis revealed increased CD11b^+^ Ly6G^high^ neutrophils in the peripheral blood of IVC-ligated mice, which was attenuated by Nec1s treatment (Supplemental Fig. 1a, b). These findings indicate that the mechanism of venous thrombus formation might involve programmed neutrophil cell death via RIPK3-MLKL signaling and histone citrullination. Next, because monocytes and macrophages produce tissue factor leading to the activation of pro-coagulant system, we evaluated the infiltration of F4/80^+^ macrophages in thrombi and the circulating CD11b^+^Ly6G^high^ monocytes. Pharmacological inhibition of RIPK1 reduced the number of infiltrating macrophages in thrombi and the circulating monocytes (Fig. 3a–c and Supplemental Fig. 1a, c). Next, to examine the role of programmed necrosis-related DAMPs during DVT formation, we measured serum histone–DNA complexes by sandwich enzyme-linked immunosorbent assay (ELISA). We observed that Nec1s treatment showed a trend toward a reduced titer of histone–DNA complexes (Supplemental Fig. 1d). These data imply that necroptotic neutrophils and NET-derived DAMPs could induce further recruitment of immune cells to the forming clot. Thus, blood cells play a critical role in the development of DVT and RIPK inhibition ameliorated venous thrombosis possibly via the suppression of neutrophil necroptosis.

### Mlkl deficiency reduces clot size in IVC thrombosis

**Fig. 4:**
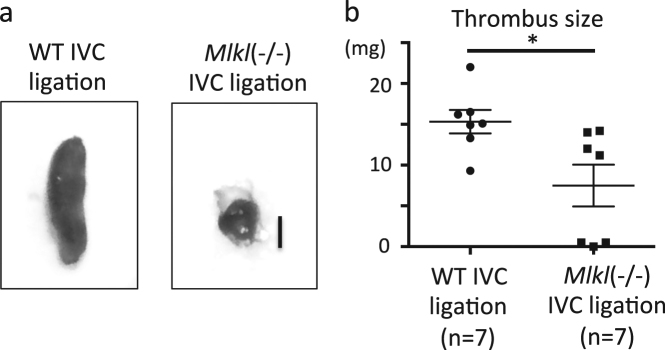
Genetic depletion of Mlkl reduces thrombus size in the IVC ligation model. **a** Macroscopic findings of thrombi in IVC-ligated wild-type (left) and *Mlkl*^*−/−*^ mice. Scale bar = 1 mm. **b** The graph shows the thrombus size of IVC-ligated wild-type (*n* = 7) and *Mlkl*^*−/−*^ mice (*n* = 7). Data are mean ± SEM in each group. *p < 0.05 vs. respective control

**Fig. 5:**
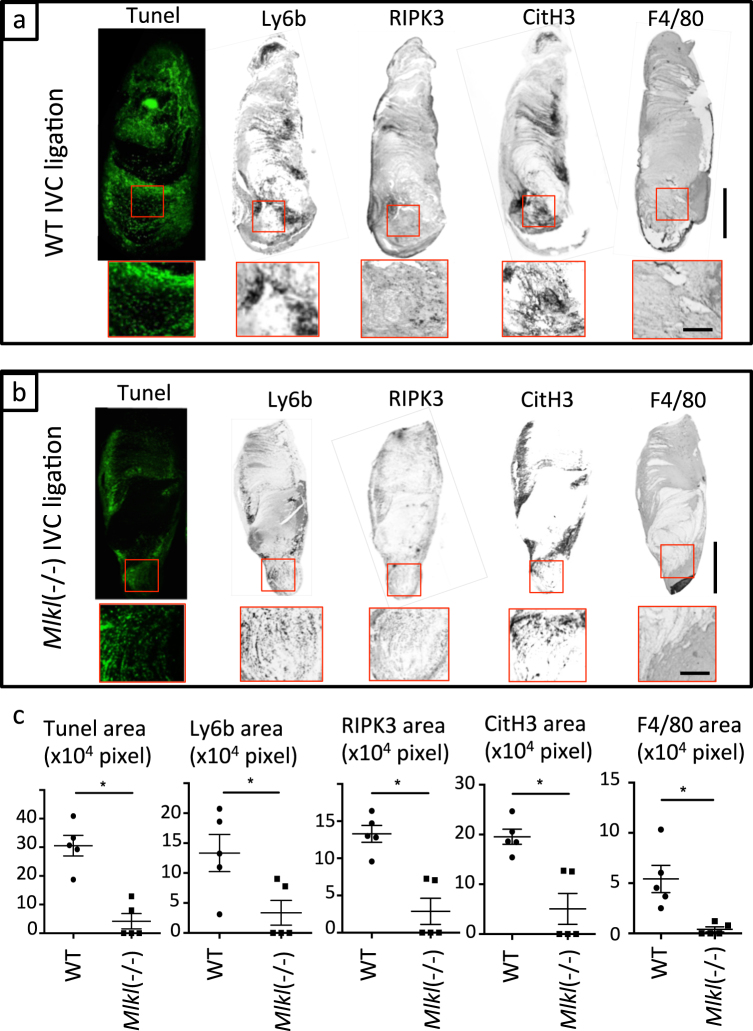
*Mlkl* gene deficiency suppresses necroptosis and NET-related signaling pathways during thrombus formation. From the left panel, TUNEL, Ly6b, RIPK3, CitH3, and F4/80 staining. **a** Representative figures of IVC-ligated wild-type mice and **b** IVC-ligated *Mlkl*^*−/−*^ mice (scale bar = 1 mm). Upper figures show the whole thrombus (scale bar = 1 mm) and lower figures show magnified image (scale bar = 250 μm). **c** Quantification of positive area of each staining. Data are mean ± SEM in each group. *p < 0.05 vs. respective control

To validate the involvement of necroptosis and also to avoid potential drug off-target effects, we applied a genetic approach using *Mlkl*-deficient mice. We observed that *Mlkl* deficiency significantly reduced clot size upon IVC ligation 3 days after surgery in mice (Fig. 4a, b). Similar to Nec1s treatment, the areas of TUNEL+ cells, Ly6b+ neutrophils, and F4/80+ macrophages in thrombi of IVC-ligated *Mlkl*-deficient mice were reduced compared to wild-type mice (Fig. 5a–c). Furthermore, the expression of RIPK3 and CitH3 in thrombi of IVC-ligated *Mlkl-*deficient mice was significantly lower compared to wild-type mice (Fig. 5a–c). In addition, *Mlkl* deficiency resulted in less circulating neutrophils, monocytes, and serum histone–DNA complexes after IVC ligation. There was no difference between groups at baseline (before surgery) in the number of neutrophils, monocytes, DAMPs, or bleeding time (Supplemental Fig. 2a–d). Taken together, *Mlkl* deficiency reduces clot size in IVC thrombosis, possibly by abrogating MLKL-dependent necroptosis of blood cells, especially neutrophils.

### Activated platelets induce neutrophil necroptosis and neutrophil–platelet aggregation

**Fig. 6:**
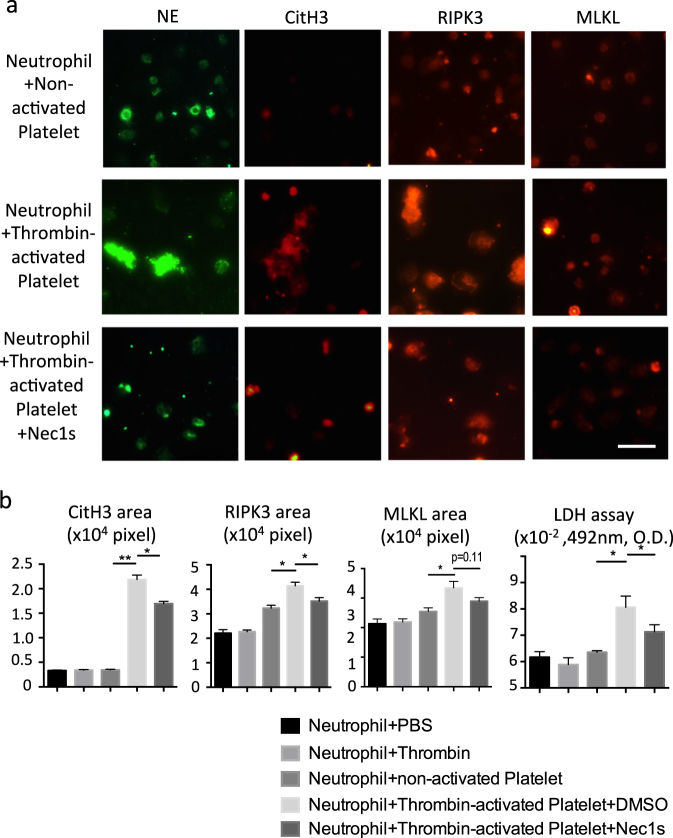
Activated platelets stimulate neutrophils leading to up-regulation of necroptosis-related and NET-related signaling molecules in vitro. **a** Upper, middle, and lower panel show co-culture images of neutrophils with non-activated platelets, neutrophils with thrombin-activated platelets, and Nec1s-treated neutrophils with thrombin-activated platelets. Immunofluorescent images show neutrophil elastase (NE): green; citrullinated histones H3 (CitH3): red; RIPK3: red; and MLKL: red. Scale bar = 50 μm. Quantification of CitH3 (**b**), RIPK3 (**c**), MLKL (**d**) area and LDH release of supernatant (**e**) in unstimulated neutrophils, thrombin-treated neutrophils, non-activated platelets, vehicle-treated neutrophils with thrombin-activated platelets, and Nec1s-treated neutrophils with thrombin-activated platelets. Data represent the mean ± SEM of three independent experiments and were analyzed using the paired *t* test. *p < 0.05 vs. respective control; **p < 0.01 vs. respective control

**Fig. 7:**
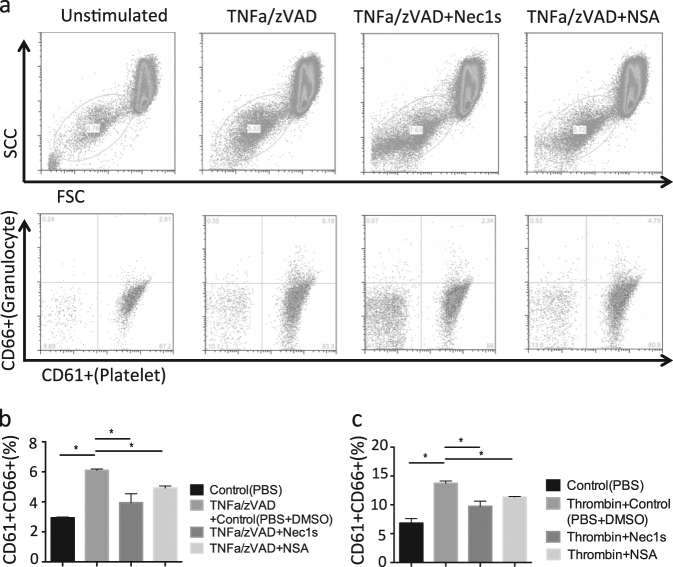
Nec1s and necrosulfonamide (NSA) inhibit aggregation between neutrophils and platelets in vitro. **a** Human whole blood was stimulated with TNFα/zVAD in the presence of vehicle, Nec1s, and NSA. Upper flow cytometry images show the platelet population gated by forward scatter (FSC)/sideward scatter (SCC). Lower images show neutrophil–platelet aggregates by CD61/CD66 gating and the aggregation ratio in TNFα/zVAD-treated (**b**) and thrombin-treated (**c**) blood. Data represent the mean ± SEM of three independent experiments and were analyzed using the paired *t* test. *p < 0.05 vs. respective control

Because blood cell necroptosis and NET formation were detected in thrombi of IVC-ligated mice, we questioned which blood cells underwent necroptosis, and how NETs are induced during thrombus formation? We first examined the expression of RIPK3 and MLKL in human neutrophils, peripheral blood mononuclear cells (PBMCs), and platelets by immunofluorescence staining as well as immunoblotting. Immunostaining revealed that neutrophils and platelets both express RIPK3 and MLKL (Supplemental Fig. 3a, b). In addition, immunoblotting analysis showed the presence of RIPK3 and MLKL protein in neutrophils, PBMCs, and platelets (Supplemental Fig. 3c). Next, we explored whether thrombin-activated platelets induce neutrophil necroptosis via the RIPK signaling pathway in vitro. Although neutrophils were not directly affected by the addition of thrombin and non-activated platelets, thrombin-activated platelets stimulated neutrophils to undergo NET formation with high expression of CitH3, RIPK3, and MLKL and with lactate dehydrogenase (LDH) release, the latter indicating neutrophil death (Fig. 6a–e). NET formation and LDH release were both suppressed by pretreatment with Nec1s (Fig. 6a–e). Considering the presence of blood cells with phosphorylated MLKL in human thrombus (Fig. 1a), the dead neutrophils undergo necroptosis. To verify that this process contributes to granulocyte–platelet aggregation as a central mechanism of clot formation, whole blood was incubated with tumor necrosis factor-α (TNFα)/zVAD known as typical inducer of necroptosis. As a readout granulocyte–platelet aggregation was analyzed by flow cytometric analysis using CD61 (platelet) and CD66 (granulocyte) markers. TNFα/zVAD induced granulocyte–platelet interaction and pretreatment with the RIPK1 inhibitor Nec1s or the MLKL inhibitor necrosulfamide (NSA) inhibited this process (Fig. 7a, b). Furthermore, as a second and more physiological inducer thrombin triggered the same interaction of granulocytes and platelets, which was rescued by Nec1s and NSA pretreatment (Fig. 7c). Finally, we examined whether platelets themselves undergo necroptosis by thrombin stimulation. The platelet death and activation in vitro was examined by flow cytometry using annexin V and P-selectin as activation markers, respectively. TNFα/zVAD-stimulated human platelets up-regulated annexin V and P-selectin; however, these phenomena were not inhibited by Nec1s and NSA (Supplemental Fig. 4a). Similarly, thrombin up-regulated annexin V and P-selectin expression in platelets, whereas Nec1s or NSA had no inhibitory effect (Supplemental Fig. 4b). Taken together, activated platelets induce neutrophil necroptosis and neutrophil–platelet aggregation.

## Discussion

**Fig. 8:**
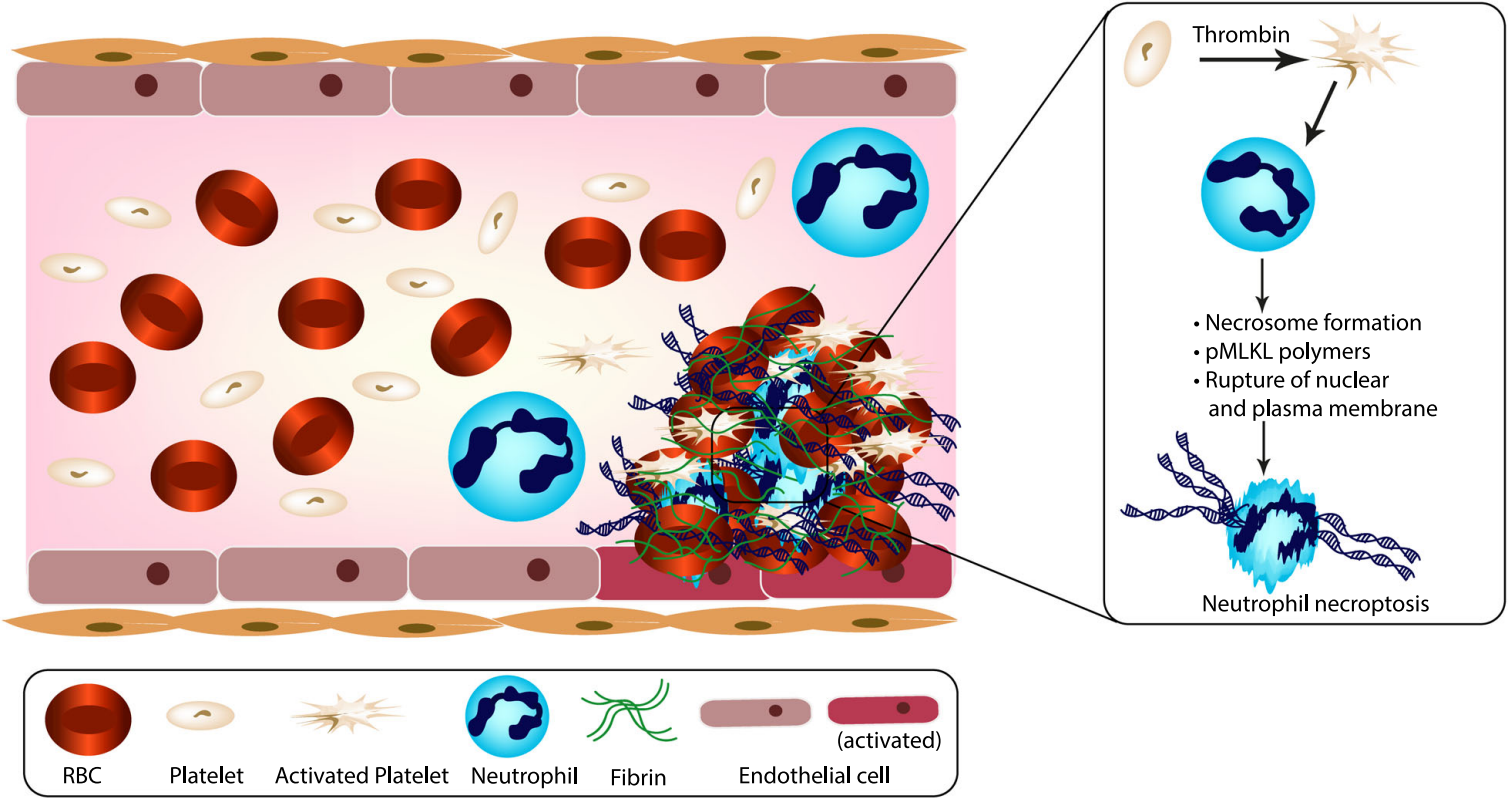
Schema of activated platelet-induced neutrophil necroptosis in DVT. During thrombus formation, RBCs initiate thrombin generation and platelet activation. The activated platelets affect neutrophils to induce neutrophil necroptosis via the phosphorylation of MLKL. Necroptotic neutrophil-derived extracellular chromatin can interact with fibrin mesh and activate endothelial cells, resulting in the aggravation of rigid clot formation as immunothrombosis. RBC red blood cell, pMLKL phosphorylated mixed lineage kinase domain-like

We had hypothesized that MLKL-dependent neutrophil necroptosis would contribute to VTE and employed specific antagonists and *Mlkl*-deficient mice to address this concept experimentally both in vitro and in vivo. We found the essential mediators of necroptosis, RIPK3 and MLKL, to be present in IVC thrombi of humans and mice. Interfering with necroptosis, either with the specific antagonist Nec1s or with genetic deletion of *Mlkl*, partially protected mice from IVC ligation-induced venous thrombosis. Mechanistically, thrombin-related platelet activation triggered neutrophil death and neutrophil-platelet aggregation, which both could be reversed by specific necroptosis inhibitors. Platelet activation itself did not involve this pathway. We, therefore, conclude that in VTE activated platelets induce neutrophil necroptosis, a process generating the release of chromatin and DAMPs that contribute to clot formation (Fig. 8).

NETs form predominantly during the organizing stage of VTE development^22^. NETs are released by neutrophils present in the lesion, and several studies identified platelet-derived HMGB1 as a trigger for NET formation^9–11,23^. Lytic proteases, histones, and DAMPs released along with NETs certainly contribute to the local and systemic inflammation associated with VTE^2,8,24,25^. However, the sticky DNA itself seems to be an essential component of the clot and synergizes with the fibrin mesh to retain red blood cells^25^. Indeed, endogenous DNases counterbalance this phenomenon in conceptually similar manner to plasmin that degrades the fibrin mesh^26^. There is an ongoing debate whether NET release is necessarily associated with neutrophil death or not. During host defense neutrophils have been shown to continue migrating also after NET release, but this has not been observed in other disease settings^16,27^. For example, in gout the exposure to urate crystals triggers crystal–NET aggregates involving lytic neutrophil death and production of a sticky creamy mass of NETs, dead neutrophils, and crystals, called the gouty tophus^28,29^. In VTE the process and results are conceptually similar, although the additional presence of blood components such as platelets, red blood cells, and the fibrin mesh produce a much higher consistency of clots vs. gouty tophi. Nevertheless, neutrophil death is essential in this process and contributes to vascular occlusion, obstructing the blood flow. Indeed, the interesting thing in the setting of VTE is that neutrophils undergo necroptosis, a form of regulated cell necrosis^19^. We recently described that urate as well as numerous other crystals and microparticles of different sizes and shapes induce neutrophil necroptosis via RIPK1, RIPK3, and MLKL^14,15^. Neutrophil necroptosis has also been observed in other conditions including exposure to granulocyte–macrophage colony-stimulating factor followed by the ligation of adhesion receptors such as CD44, CD11b, CD18, or CD15^30^. Our data add thrombin-activated platelets to the potential triggers of neutrophil necroptosis, although the precise outside-in signaling mechanism remains to be determined. We also do not claim that the process of neutrophil necroptosis is identical to that of NET release or what has been called “NETosis.”^21^ However, the consequence of plasma cell rupture in neutrophil necrosis is the same, that is, sticky NET-like chromatin immobilizes adjacent particles, which in VTE are red blood cells, platelets, and the fibrinogen mesh^2^.

Interestingly, we found that platelets also express RIPK3 and MLKL. These are ubiquitous cytoplasmic proteins, which are obviously shed from megakaryocytes during platelet formation. Although platelet necrosis is known to be regulated by mitochondrial effect with calcium reflux and ATP depletion^31^, how necroptosis contributes to the platelet death remains unknown. It has been reported that deletion of RIPK3 from megakaryocytes and platelets causes a marked defect in platelet aggregation and attenuates dense granule secretion in response to thrombin or a thromboxane A2 analog in vitro, and delay vascular occlusion time in a mouse model of arterial thrombosis^13^. It should be noted that RIPK3 has promiscuous biological functions beyond necroptosis, for example, in apoptosis or interleukin-1-dependent or nuclear factor-κB-dependent inflammation^12^, as well as platelet activation^13^. Nevertheless, we did not find any evidence that MLKL inhibition affects thrombin-induced platelet activation. Therefore, we consider the VTE phenotype of *Mlkl*-deficient mice largely relate to the lack of neutrophil necroptosis. Unfortunately, *Mlkl*^*flox*^ mice were not accessible to us to study cell-type-specific deletion of MLKL.

In summary, in VTE activated platelets, and possibly other triggers, induce neutrophil necroptosis, a process generating the release of chromatin and DAMPs that contribute to clot formation. Thus, inhibitors of necroptosis may interfere with clotting, which might be explored for therapeutic purposes.

## Materials and methods

### Venous thrombosis model

One hundred percent flow obstruction of the IVC was induced in 12- to 13-week-old male wild-type C57BL/6N mice (Charles River Laboratories, Sulzfeld, Germany) or *Mlkl*^*−*^^/*−*^ mice under the maintenance of normal body temperature by employing preoperative heat supply and online core body temperature recording as described^32^. Mice were anesthetized by intraperitoneal injection of medetomidine (0.5 mg/kg), midazolam (5 mg/kg), and fentanyl (0.05 mg/kg) before median laparotomy was performed to carefully expose and completely ligate the IVC using 7-0 prolene (ETHICON) exactly below the left renal vein without manipulating side branches. After ligation, the abdominal wall and skin were closed by sutures. Anesthesia was antagonized by subcutaneous injection of atipamezol 2.5 mg/kg and flumazenil 0.5 mg/kg and pain control was assured by regular subcutaneous injections of buprenorphine 1 mg/kg every 8 h. Mice with surgical complications such as bleeding or injury of the IVC were excluded because these factors could possibly affect thrombus formation. Other groups of C57BL/6N wild-type mice were treated with Nec1s (1.65 mg/kg, intraperitoneally, Bio Vision, USA) or vehicle (10% dimethyl sulfoxide (DMSO) in phosphate-buffered saline (PBS)) 1 h before the surgery. All mice were sacrificed 3 days after surgery and thrombus weight (without vessels) was measured as a primary endpoint of clot formation. As a bleeding test 10- to 12-week-old male C57BL/6N wild-type or *Mlkl*^−/−^ mice were anesthetized using isofluorane. A 2 mm segment of the tail tip was cut using a scalpel, and the tail was put in 37 °C PBS^33^. Bleeding time was recorded up to when bleeding had completely stopped. All animal-related procedures fulfilled the directive 2010/63/EU and were optimized in terms of 3R recommendations and approved by the local governmental authorities (ROB-55.2Vet-2532.Vet_02-17-54).

### Histological examination

Thrombi were embedded in paraffin and 3 μm sections were deparaffinized and rehydrated as previously described^34^. Sections were stained with H&E or prepared for immunohistochemistry. A 0.3% H_2_O_2_ was used for inhibition of endogenous peroxidase. Primary antibodies included rat anti-mouse Ly6b (neutrophils, AbD Serotec, Oxford, UK), rabbit anti-CitH3 (netting neutrophils, Abcam, Cambridge, UK), rabbit anti-mouse RIPK3 (Abcam, Cambridge, UK), anti-mouse MLKL (kindly provided by Andreas Linkermann, Dresden), and rat anti-mouse F4/80 (Serotec, Oxford, UK)^35^. TUNEL staining kit (Roche, Mannheim, Germany) was used to detect dying cells inside the thrombus following the manufacturer’s description. Positive cells were quantified using the ImageJ software.

### Histological examination in human thrombus

Paraffin-embedded sections of a human IVC thrombus from a patient with renal cell carcinoma were stained with H&E and immunohistochemistry was performed using the following primary antibodies rabbit anti-CD61 (LifeSpan Biosciences, Inc. Seattle, WA, USA), rabbit anti-fibrinogen, rabbit anti-myeloperoxidase, rabbit anti- RIPK3, rabbit anti-phosphorylated MLKL (all from Abcam, Cambridge, UK). Signal detected was performed using routine procedures as described^36^.

### In vitro experiments

Blood was obtained from healthy donors after providing written informed consent on forms approved by the “Ethikkommission der Medizinischen Fakultät der LMU” and all experiments were performed in accordance with their guidelines and regulations. Neutrophils were isolated using standard dextran sedimentation followed by Ficoll–Hypaque density centrifugation procedures^14,15^. For platelet isolation, blood was collected into sodium citrate-coated tubes. Platelet-rich plasma was obtained by centrifugation (200 rpm, 20 min). Neutrophils were suspended in RPMI (5 × 10^5^ cells/well), and seeded onto either eight-well micro-slides (Ibidi, Martinsried, Germany) or 96-well plates, and incubated in a 5% carbon dioxide atmosphere at 37 °C for 30 min. Neutrophils were pre-treated with Nec1s (100 µM, Enzo, Lörrach, Germany) or vehicle (1% DMSO in PBS) for 30 min and then stimulated with thrombin (0.05 U/ml, Merck Millipore, Darmstadt, Germany), non-activated platelets, and thrombin (0.05 U/ml for 3 min) activated platelets (1 × 10^7^ cells/well). After 3-h incubation, the microslides were fixed with 4% paraformaldehyde (PFA) and analyzed for CitH3, RIPK3, and MLKL expression by immunofluorescence staining. In 96 plates, neutrophil death was quantified by the LDH assay (Sigma Aldrich, Steinheim, Germany) using neutrophil supernatants. To induce neutrophil–platelet aggregation, human whole blood was stimulated by either the combination TNFα (200 ng/ml) and zVAD (20 µM) or 0.05 U/ml thrombin (for 10 min) with or without pretreatment of Nec1s (100 µM) and necrosulfonamide (10 µM, Calbiochem).

### Flow cytometric analysis

Flow cytometric analysis was performed on a FACS Calibur flow cytometer (BD Biosciences). In mouse experiments, anti-mouse FITC-Ly6G, PerCP-Ly6C, PE-CD11b (BD Biosciences), and APC-CD45 (BioLegend) antibodies were used to identify circulating neutrophils and activated monocytes in peripheral blood. Mouse plasma was analyzed for histone–nucleosome complexes by ELISA (Roche). Whole blood was analyzed by flow cytometry to quantify circulating immune cells^36^. In vitro, human platelets and whole blood were used. Platelets were gated by APC-CD42b (BioLegend) and platelet activation and death were determined by PerCP-CD62p (BioLegend) and FITC-Annexin V (BD Pharmingen), respectively. Platelet–granulocyte aggregation was determined using anti-human FITC-CD61 and PE-CD66 (BioLegend) antibody in accordance with the manufacturer’s instructions.

### Histone-nucleosome assay

Serum histone was evaluated by histone–DNA complexes ELISA kit (Roche, Mannheim, Germany).

### Immunoblotting

Blood cells were also analyzed by standard immunoblotting. Cell pellets were lysed with RIPA buffer (Sigma, USA), the extracted proteins were separated by sodium dodecyl sulfate-polyacrylamide gel electrophoresis, and transferred to a polyvinylidene difluoride membrane. Anti-β-actin, RIPK3, and MLKL antibodies (Abcam, UK) were used for detection of molecules expression.

### Statistics

Data are presented as mean ± SEM. Unpaired Student’s *t* test and one-way analysis of variance followed by Dunnett’s post test were used for the comparison. A value of *p* < 0.05 was considered to indicate statistical significance.

## Electronic supplementary material

Supplemental Figure

## References

[1] Stone J (2017). Deep vein thrombosis: pathogenesis, diagnosis, and medical management. Cardiovasc. Diagn. Ther..

[2] Wolberg AS (2015). Venous thrombosis. Nat. Rev. Dis. Prim..

[3] Gando S, Levi M, Toh CH (2016). Disseminated intravascular coagulation. Nat. Rev. Dis. Prim..

[4] Cines DB (2014). Clot contraction: compression of erythrocytes into tightly packed polyhedra and redistribution of platelets and fibrin. Blood.

[5] Van Der Meijden PE (2012). Platelet- and erythrocyte-derived microparticles trigger thrombin generation via factor XIIa. J. Thromb. Haemost..

[6] Simanek R (2010). High platelet count associated with venous thromboembolism in cancer patients: results from the Vienna Cancer and Thrombosis Study (CATS). J. Thromb. Haemost..

[7] Engelmann B, Massberg S (2013). Thrombosis as an intravascular effector of innate immunity. Nat. Rev. Immunol..

[8] Papayannopoulos V (2018). Neutrophil extracellular traps in immunity and disease. Nat. Rev. Immunol..

[9] von Bruhl ML (2012). Monocytes, neutrophils, and platelets cooperate to initiate and propagate venous thrombosis in mice in vivo. J. Exp. Med..

[10] Stark K (2016). Disulfide HMGB1 derived from platelets coordinates venous thrombosis in mice. Blood.

[11] Dyer MR (2018). Deep vein thrombosis in mice is regulated by platelet HMGB1 through release of neutrophil-extracellular traps and DNA. Sci. Rep..

[12] Orozco S, Oberst A (2017). RIPK3 in cell death and inflammation: the good, the bad, and the ugly. Immunol. Rev..

[13] Zhang Y (2017). Receptor-interacting protein kinase 3 promotes platelet activation and thrombosis. Proc. Natl. Acad. Sci. USA.

[14] Desai J (2017). Particles of different sizes and shapes induce neutrophil necroptosis followed by the release of neutrophil extracellular trap-like chromatin. Sci. Rep..

[15] Desai J (2016). PMA and crystal-induced neutrophil extracellular trap formation involves RIPK1-RIPK3-MLKL signaling. Eur. J. Immunol..

[16] Wang X, Yousefi S, Simon HU (2018). Necroptosis and neutrophil-associated disorders. Cell Death Dis..

[17] Linkermann A, Green DR (2014). Necroptosis. N. Engl. J. Med..

[18] Mulay SR (2016). Cytotoxicity of crystals involves RIPK3-MLKL-mediated necroptosis. Nat. Commun..

[19] Weinlich R, Oberst A, Beere HM, Green DR (2017). Necroptosis in development, inflammation and disease. Nat. Rev. Mol. Cell. Biol..

[20] Gong YN (2017). ESCRT-III acts downstream of MLKL to regulate necroptotic cell death and its consequences. Cell.

[21] Desai J, Mulay SR, Nakazawa D, Anders HJ (2016). Matters of life and death. How neutrophils die or survive along NET release and is **“**NETosis**”** = necroptosis?. Cell. Mol. Life Sci..

[22] Savchenko AS (2014). Neutrophil extracellular traps form predominantly during the organizing stage of human venous thromboembolism development. J. Thromb. Haemost..

[23] Brill A (2012). Neutrophil extracellular traps promote deep vein thrombosis in mice. J. Thromb. Haemost..

[24] Allam R, Kumar SV, Darisipudi MN, Anders HJ (2014). Extracellular histones in tissue injury and inflammation. J. Mol. Med. (Berl.).

[25] Kimball AS, Obi AT, Diaz JA, Henke PK (2016). The emerging role of NETs in venous thrombosis and immunothrombosis. Front. Immunol..

[26] Jimenez-Alcazar M (2017). Host DNases prevent vascular occlusion by neutrophil extracellular traps. Science.

[27] Yipp BG (2012). Infection-induced NETosis is a dynamic process involving neutrophil multitasking in vivo. Nat. Med..

[28] Schauer C (2014). Aggregated neutrophil extracellular traps limit inflammation by degrading cytokines and chemokines. Nat. Med..

[29] Desai J, Steiger S, Anders HJ (2017). Molecular pathophysiology of gout. Trends Mol. Med..

[30] Wang X, He Z, Liu H, Yousefi S, Simon HU (2016). Neutrophil necroptosis is triggered by ligation of adhesion molecules following GM-CSF priming. J. Immunol..

[31] Jackson SP, Schoenwaelder SM (2010). Procoagulant platelets: are they necrotic?. Blood.

[32] Marschner JA, Schafer H, Holderied A, Anders HJ (2016). Optimizing mouse surgery with online rectal temperature monitoring and preoperative heat supply. Effects on post-ischemic acute kidney injury. PLoS ONE.

[33] Gushiken FC, Han H, Li J, Rumbaut RE, Afshar-Kharghan V (2009). Abnormal platelet function in C3-deficient mice. J. Thromb. Haemost..

[34] Nakazawa D (2017). Histones and neutrophil extracellular traps enhance tubular necrosis and remote organ injury in ischemic AKI. J. Am. Soc. Nephrol..

[35] Lech M (2014). Macrophage phenotype controls long-term AKI outcomes—kidney regeneration versus atrophy. J. Am. Soc. Nephrol..

[36] Patole PS (2007). Coactivation of Toll-like receptor-3 and -7 in immune complex glomerulonephritis. J. Autoimmun..

