# Supplementary material for: Activated platelets induce MLKL-driven neutrophil necroptosis and release of neutrophil extracellular traps in venous thrombosis
Source: Cell Death Discov. 2018 Jun 28;4:71. doi: 10.1038/s41420-018-0073-2 (PMC6060161; doi:10.1038/s41420-018-0073-2)
Supplement: Supplementary file 1 — Supplemental Figure [file 41420_2018_73_MOESM1_ESM.pdf]

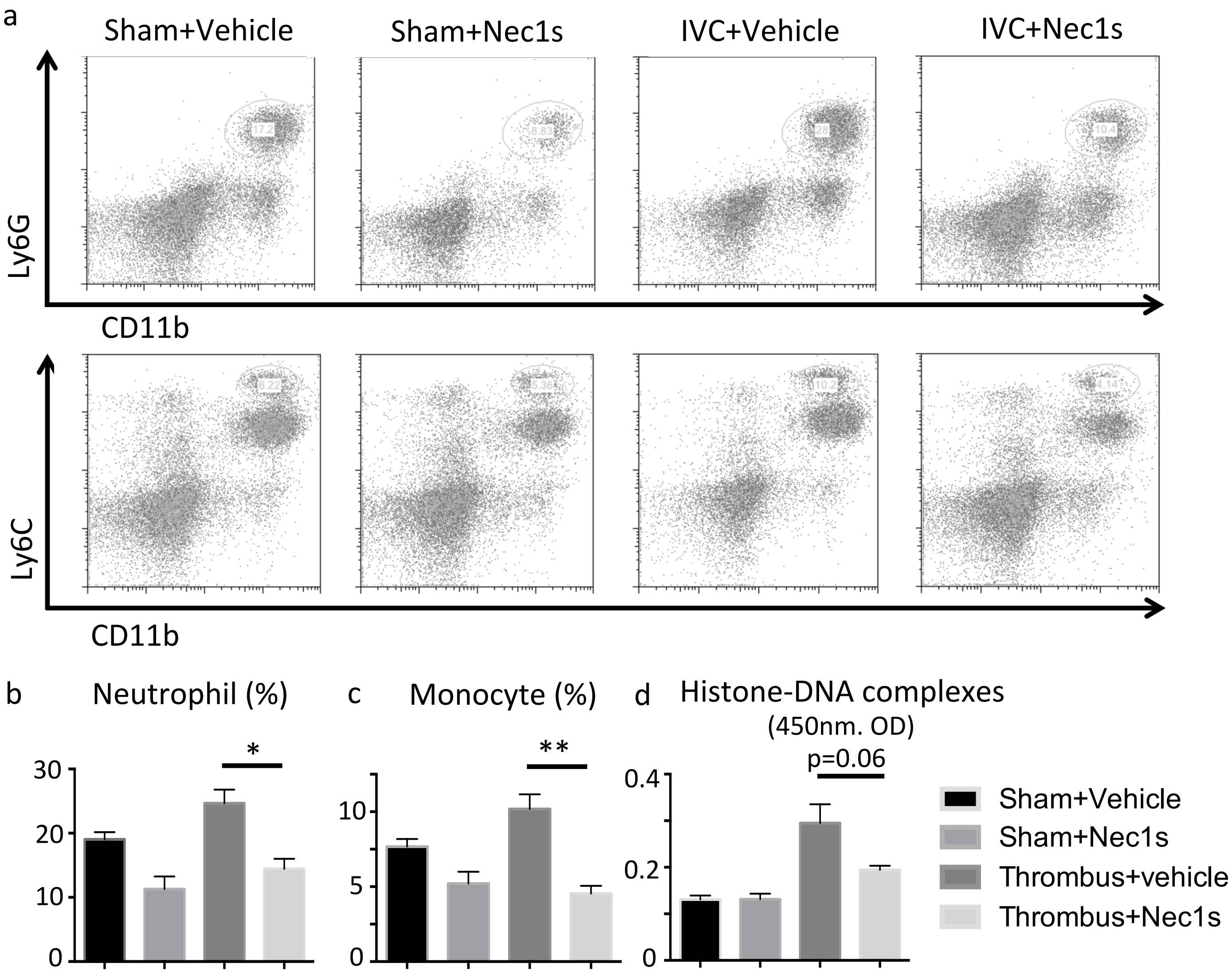

Supplemental figure1. Nec1s reduces the circulating inflammatory cells and histone during thrombus formation. The whole blood of sham or IVC ligation-operated mice was obtained 3 days after the surgery. a) Dot-plots image from flow cytometry analysis to illustrate the gating to obtain the population of CD11b+/Ly6G+ neutrophils (upper figures) and CD11b+/Ly6c^{high} monocytes (lower figures). The percentage of neutrophils (b) and monocytes (c) of CD45-gated blood cells. (d) Serum histones were evaluated with histone-DNA complexes ELISA kit. Data are mean ± SEM in each group. *p<0.05 versus respective control; **p<0.01 versus respective control.

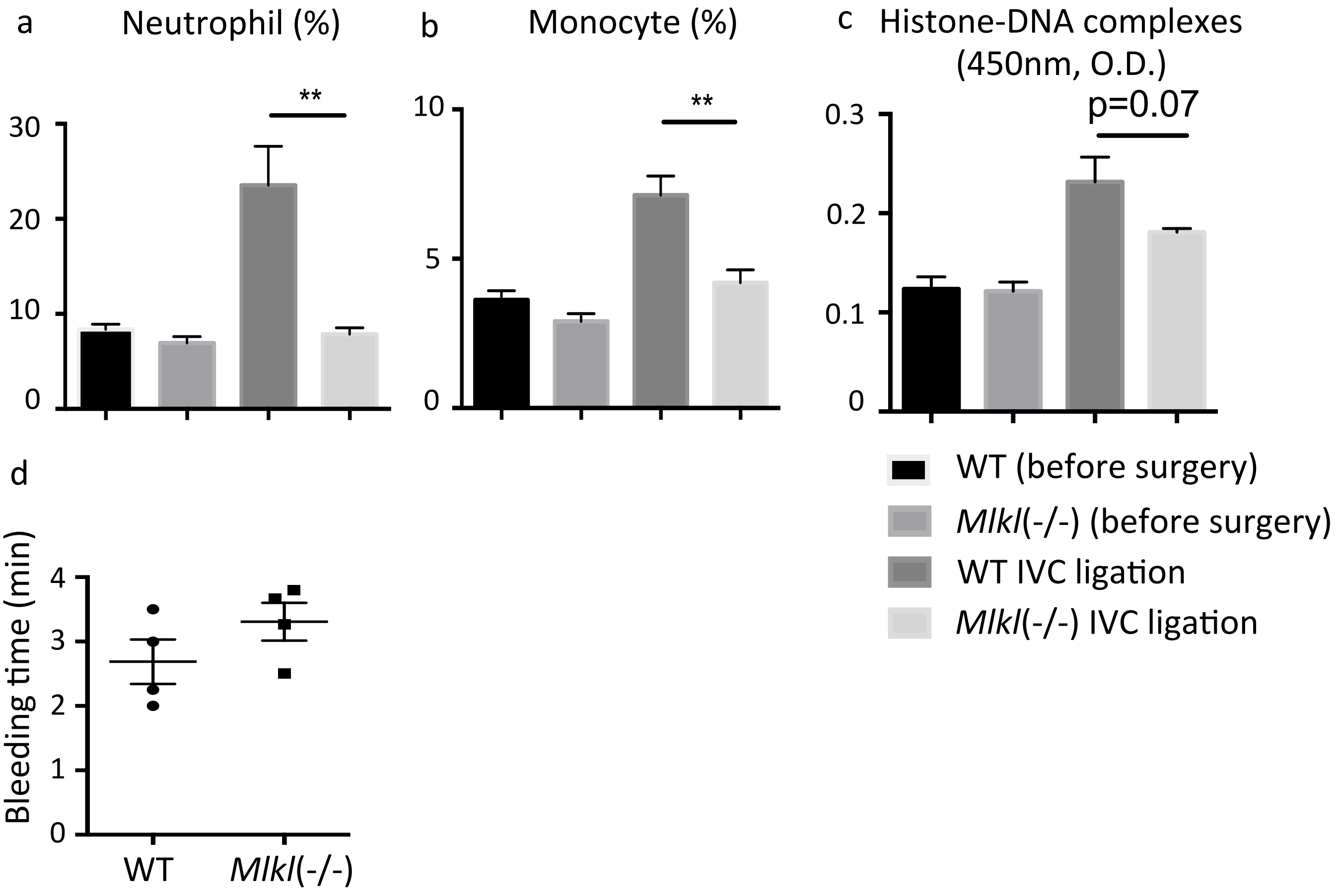

Supplemental figure 2. *Mlkl* gene deficiency reduces the circulating inflammatory cells and histones during thrombus formation. The percentage of CD11b+/Ly6G+ neutrophils (a) and CD11b+/Ly6c^{high} monocytes (b) of CD45-gated blood cells in wildtype and *Mlkl-/-* mice at before and 3 days after IVC ligation surgery. c) The level of serum histones. d) Bleeding time test in wild type and *Mlkl-/-* mice before the surgery. Data are mean ± SEM in each group. *p<0.05 versus respective control; **p<0.01 versus respective control.

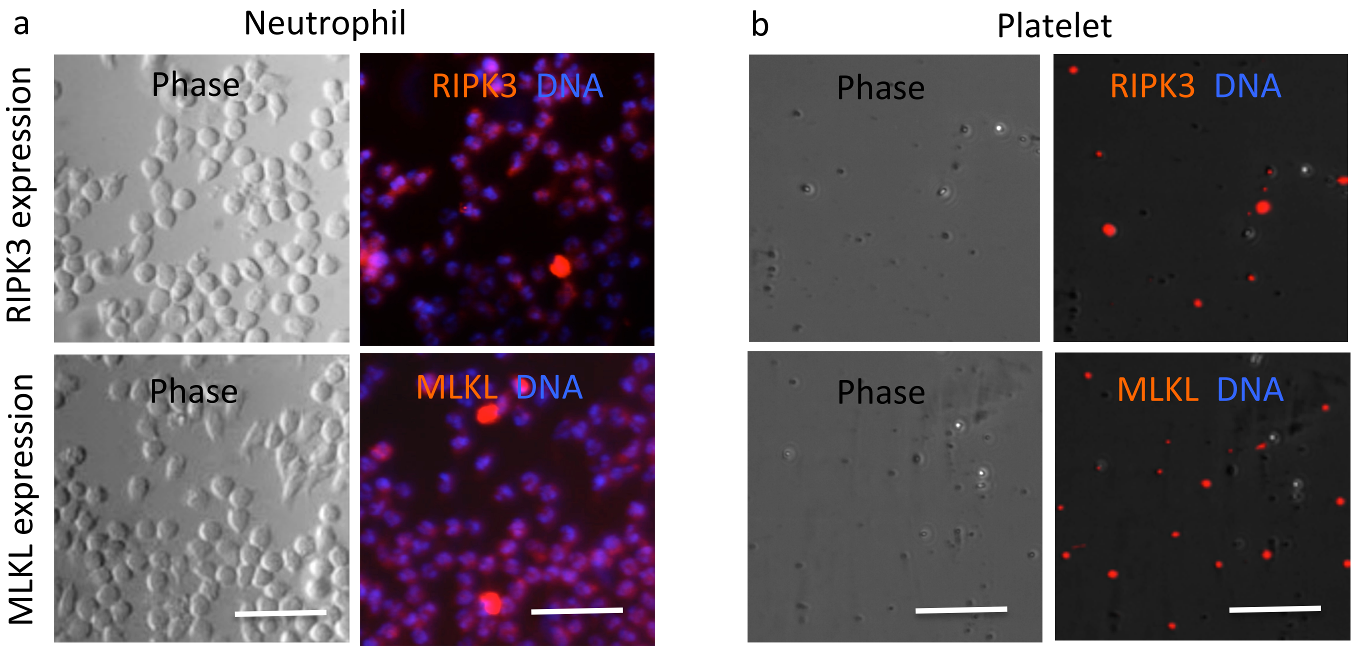

Supplemental figure 3. The expression of necroptosis-related molecules in blood cells. Human neutrophils (a) and platelets (b) express the RIPK3 and MLKL. Left figures show phase contrast images. Right upper figure shows immunofluorescence staining of RIPK3 (red), right lower figure shows MLKL (red) and 49,6-diamidin-2-phenylindol (DAPI) staining (blue). Scale bar=50µm. c) The expression of RIPK3 and MLKL protein in neutrophils, peripheral blood mononuclear cells and platelets were detected by immunoblotting with β-actin as a loading control.

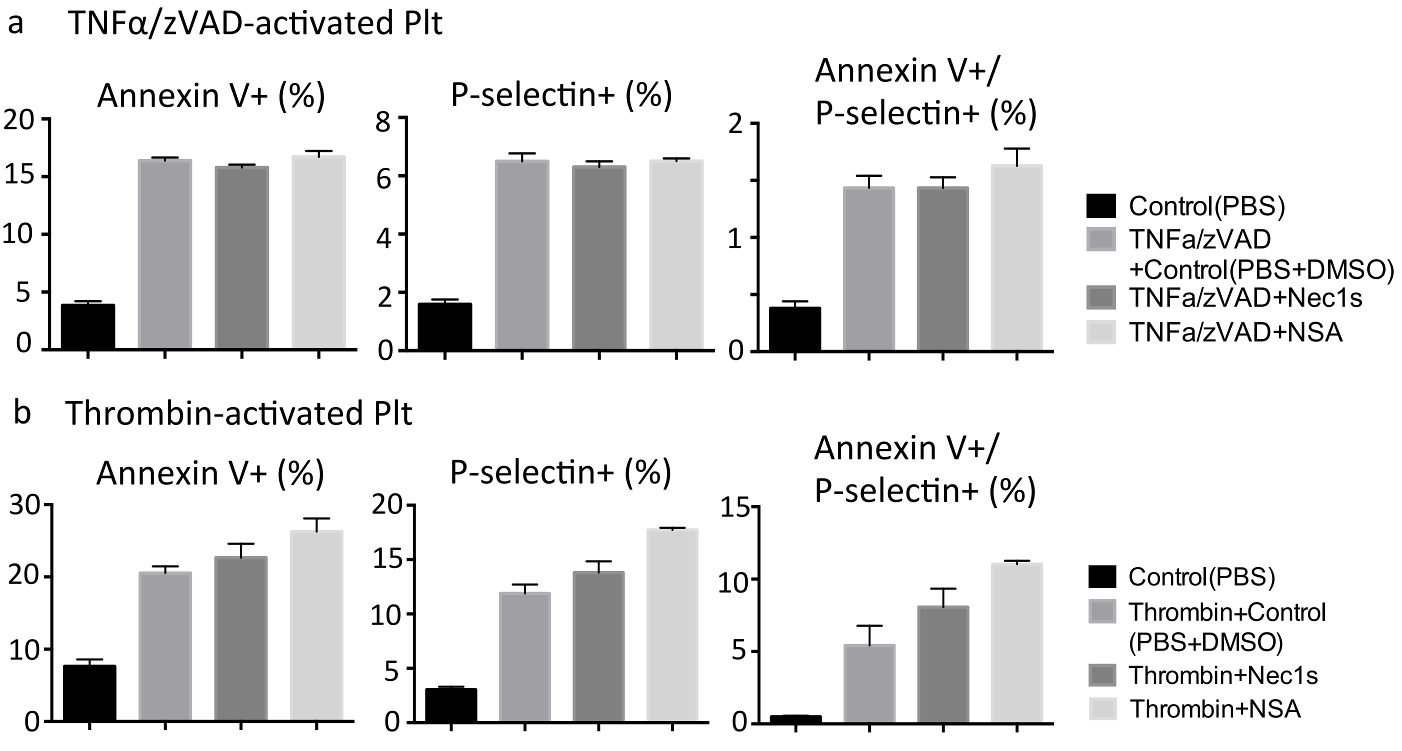

Supplemental figure 4. Nec1s and NSA do not suppress platelet activation or death. Human platelets were stimulated with TNFα/zVAD (a) and thrombin in the presence of vehicle, Nec1s or NSA. The activated and dead platelets were analyzed by annexin V and P-selection expression. Data represent the mean ± SEM of three independent experiments and were analyzed using the paired t test.
